# A *Genomic Encyclopedia of the Root Nodule Bacteria*: assessing genetic diversity through a systematic biogeographic survey

**DOI:** 10.1186/1944-3277-10-14

**Published:** 2015-02-09

**Authors:** Wayne Reeve, Julie Ardley, Rui Tian, Leila Eshragi, Je Won Yoon, Pinyaruk Ngamwisetkun, Rekha Seshadri, Natalia N Ivanova, Nikos C Kyrpides

**Affiliations:** 1 Centre for Rhizobium Studies, Murdoch University, Murdoch, Western Australia, Australia; 2 Centre for Phytophthora Science and Management, Murdoch University, Murdoch, Western Australia, Australia; 3 DOE Joint Genome Institute, Walnut Creek, CA, USA; 4 Department of Biological Sciences, King Abdulaziz University, Jeddah, Saudi Arabia

**Keywords:** GEBA-RNB, Root nodule bacteria, Diversity, Symbiosis, Nitrogen fixation

## Abstract

Root nodule bacteria are free-living soil bacteria, belonging to diverse genera within the *Alphaproteobacteria* and *Betaproteobacteria*, that have the capacity to form nitrogen-fixing symbioses with legumes. The symbiosis is specific and is governed by signaling molecules produced from both host and bacteria. Sequencing of several model RNB genomes has provided valuable insights into the genetic basis of symbiosis. However, the small number of sequenced RNB genomes available does not currently reflect the phylogenetic diversity of RNB, or the variety of mechanisms that lead to symbiosis in different legume hosts. This prevents a broad understanding of symbiotic interactions and the factors that govern the biogeography of host-microbe symbioses.

Here, we outline a proposal to expand the number of sequenced RNB strains, which aims to capture this phylogenetic and biogeographic diversity. Through the Vavilov centers of diversity (Proposal ID: 231) and GEBA-RNB (Proposal ID: 882) projects we will sequence 107 RNB strains, isolated from diverse legume hosts in various geographic locations around the world. The nominated strains belong to nine of the 16 currently validly described RNB genera. They include 13 type strains, as well as elite inoculant strains of high commercial importance. These projects will strongly support systematic sequence-based studies of RNB and contribute to our understanding of the effects of biogeography on the evolution of different species of RNB, as well as the mechanisms that determine the specificity and effectiveness of nodulation and symbiotic nitrogen fixation by RNB with diverse legume hosts.

## Introduction

### The importance of the research

Legumes, with around 20,000 species and over 700 genera, are the third largest flowering plant family and are found on all continents (except Antarctica). They are major components of most of the world’s vegetation types and have important roles in agriculture as both pastures and pulses [1,2]. Most legumes are able to form dinitrogen-fixing symbioses with soil bacteria, collectively known as root nodule bacteria or rhizobia. RNB infection elicits the organogenesis of a unique structure, the nodule, which forms on the root (or less commonly, the stem) of the host plant. The mode of infection and the morphology and structure of the resulting nodule varies within the different legume tribes and has phylogenetic significance [3,4]. Following infection, RNB migrate to the nodule primordium, are endocytosed within the host cell and differentiate into N_2_-fixing bacteroids.

The availability of utilizable nitrogen is the critical determinant for plant productivity. Legume-RNB symbiotic nitrogen fixation is a vital source of N in both natural and agricultural ecosystems. Based on different estimates, the total annual input of biologically fixed N ranges from 139 to 175 million tons, 35 to 44 million tons of which is attributed to RNB-legume associations growing on arable land, with those in permanent pastures accounting for another 45 million tons of N. N_2_-fixation by legume pastures and crops provides 65% of the N currently utilized in agricultural production [5,6]. The economic value of legumes on the farm is estimated at $30 billion annually, including $22 billion in the value of legume crops and $8 billion in the value of N_2_-fixation. Increasing the efficiency of the legume-RNB symbiosis has been projected to have an annual US benefit of $1,067 million, while transferring SNF technology to cereals and totally eliminating chemical N fertilization of the major crops will have an annual US benefit of $4,484 million [7].

Incorporating SNF in agricultural systems also reduces energy consumption, compared with systems that rely on chemical N-input. Every ton of manufactured N-fertilizer requires 873 m^3^ of natural gas and ultimately releases ~2 tons of CO_2_ into the air [8]. Furthermore, >50% of US N-fertilizer is imported, which further increases the energy cost of chemical N fertilizer. SNF has the potential to reduce the application of manufactured N-fertilizer by ~160 million tons pa, equating to a reduction of 270 million tons of coal or equivalent fossil fuel consumed in the production process. As well as energy cost savings, this reduces CO_2_ greenhouse gas emissions. Legume- and forage-based rotations also reduce CO_2_ emission by maintaining high levels of soil organic matter, thus enhancing both soil fertility and carbon storage in soil [9]. There are additional significant environmental costs to the use of N fertilizer: agriculturally based increases in reactive N are substantial and widespread, and lead to losses of biological diversity, compromised air and water quality, and threats to human health [10]. Microbial nitrification and denitrification of soil N are major contributors to emissions of the potent greenhouse gas and air pollutant, nitrous oxide, from agricultural soils [5]. Emission of N_2_O is in direct proportion to the amount of fertilizer applied. In addition, fertilizer N not recovered by the crop rapidly enters surface and groundwater pools, leading to drinking water contamination, and eutrophication and hypoxia in aquatic ecosystems [8].

The global increase in population is predicted to double demand for agricultural production by 2050 [11]. To meet this demand without incurring the high and unsustainable costs associated with the increased use of chemical N-fertilizer, the N_2_-fixing potential of the legume-RNB symbiosis must be maximized. Achieving this target will require a greater understanding of the molecular mechanisms that govern specificity and effectiveness of N_2_-fixation in diverse RNB-legume symbioses.

Genome sequencing of RNB strains has revolutionized our understanding of the bacterial functional genomics that underpin symbiotic interactions and N_2_-fixation. However, previous RNB sequencing projects have not reflected the phylogenetic and biogeographic diversity of RNB or the variety of mechanisms that lead to symbiosis in different legume hosts. As a result, the insights gained into SNF have been limited to a small group of symbioses and there has not yet been a systematic effort to remedy this narrow focus.

Here, we outline proposals for two sequencing projects to be undertaken at the DoE Joint Genome Institute that aim to expand the number of sequenced RNB strains in order to capture this phylogenetic and biogeographic diversity. Through the Vavilov centers of diversity (Proposal ID: 231) and GEBA-RNB (Proposal ID: 882) projects we will sequence 107 RNB strains isolated from diverse legume hosts in various geographic locations in over 30 countries around the world. The sequenced strains belong to nine of the 16 validly described RNB genera and have been isolated from 69 different legume species, representing 39 taxonomically diverse genera, growing in diverse biomes. These proposals will provide unprecedented perspectives on the evolution, ecology and biogeography of legume-RNB symbioses, as no rhizobial sequencing project so far has attempted to relate extensive genomic characterization of RNB strains to comprehensive metadata and thereby identify correlations between the genomes of rhizobial strains, their symbiotic associations with specific legume hosts, and the environmental parameters of their habitats.

## Project design

### Selection of target organisms

The proposed RNB genome sequencing projects were designed with two different but complementary objectives in mind. In the “Analysis of the clover, pea/bean and lupin microsymbiont genetic pool by studying isolates from distinct Vavilov centres of diversity” project (Proposal ID: 231), the nominated RNB included clover, pea/*Vicia* and lupin-nodulating strains, chosen because their hosts are of highly significant commercial importance [12]. The legumes originate from six distinct Vavilov centres of diversity: the Mediterranean basin, high altitude Temperate Europe, North America, South America, highland central Africa and southern Africa [13]. The rhizobial associations in these centers have phenological and geographic specificity for nodulation and nitrogen fixation [14,15]. A detailed analysis of strains representing the six centres of diversity will enable the investigation of the evolution and biodiversity of symbioses from a geographic and phenological viewpoint.

The GEBA-RNB project falls under the umbrella of the Genomic Encyclopedia of Bacteria and *Archaea* family projects. The original GEBA project [16] sequenced and analysed the genomes of *Bacteria* and *Archaea* species selected to maximize phylogenetic coverage. RNB are polyphyletic, belonging to diverse genera of the *Alphaproteobacteria*- and *Betaproteobacteria*; currently, 16 genera and over 100 species have been validly described (ICSP Subcommittee on the taxonomy of *Rhizobium* and *Agrobacterium*). Existing RNB sequencing programs have tended to focus on particular organisms or on RNB isolated from specific hosts. The GEBA-RNB project was therefore designed as a systematic genome sequencing project to capture RNB phylogenetic and symbiotic diversity. RNB strains were selected on the basis of (i) phylogenetic diversity, (ii) legume host diversity, (iii) economic importance and (iv) biogeographic origin. Strains were also required to have comprehensive metadata records and well characterized phenotypes, in particular relating to symbiotic effectiveness. In addition, the phylogenetic divergence of strains from previously sequenced isolates was taken into account.

**Figure 1:**
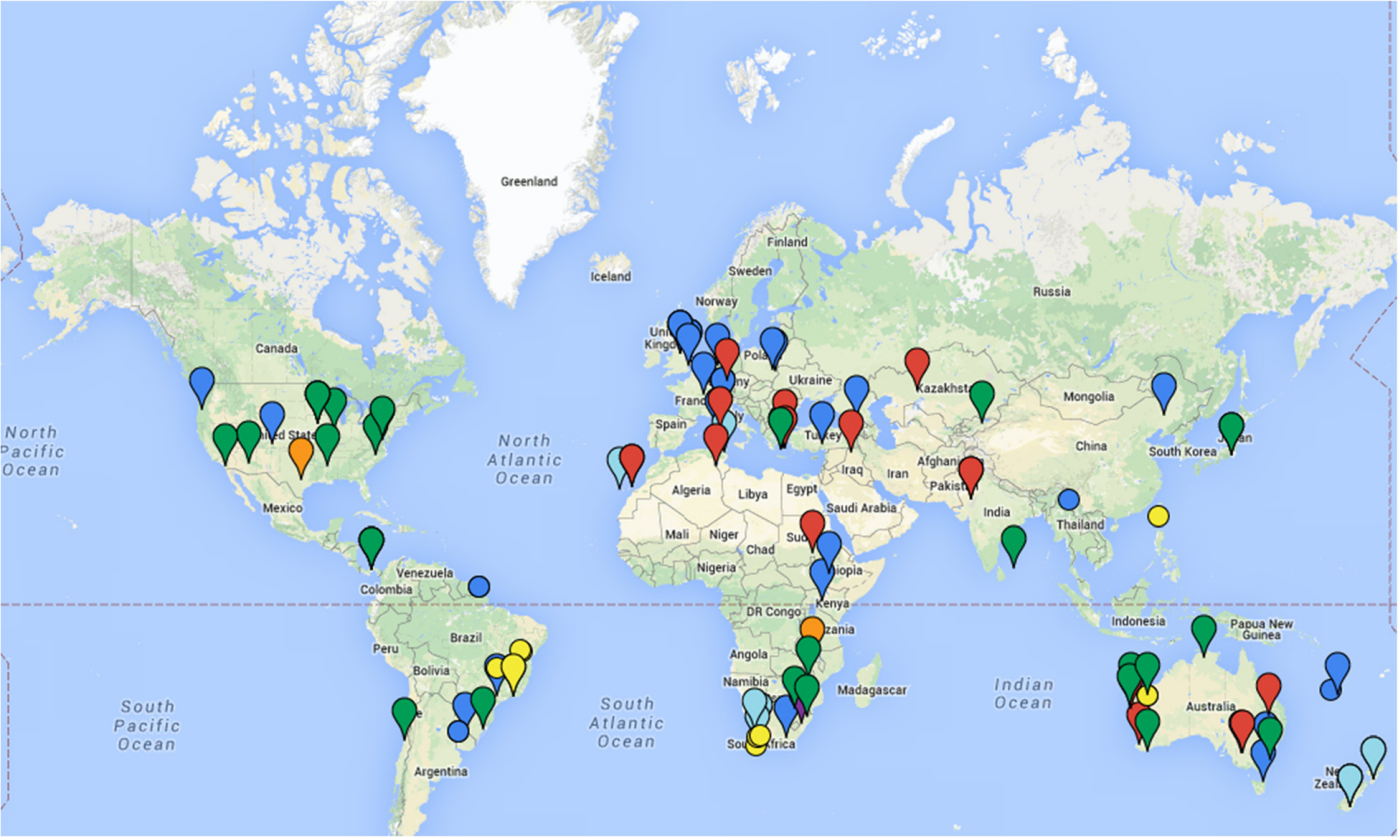
**This map shows the location of isolation of root nodule bacteria strains requested for sequencing in this proposal.** Color codes are as follows: *Alphaproteobacteria*; *Azorhizobium* (yellow), *Bradyrhizobium* (green), *Ensifer* (red), *Mesorhizobium* (light blue), *Methylobacterium* (purple), *Microvirga* (orange), *Rhizobium* (blue) and *Betaproteobacteria* (circles); *Burkholderia* (yellow), *Cupriavidus* (blue).

The map in Figure 1 shows collection sites of strains selected for sequencing. Table 1 lists the strains nominated for sequencing, their country of origin and original host. Extensive metadata is available for all strains and was used to guide strain selection; proposed strains display a wide range of host specificities (from strictly specific to highly promiscuous) and SNF efficiency. The RNB were collected from sites that spanned a broad range of soils and climates (e.g. neutral, acidic or alkaline soil, tropical, arid or temperate climate). These strains differ in their physiological attributes (ability to recycle hydrogen, rhizobitoxine production, salt and acid tolerance, heavy metal resistance, methylotrophy) and some of them display unusual genetic features (unique genotype based on multilocus sequence typing, nodulation phenotype, atypical organization of symbiosis islands or identical symbiosis islands in different genetic backgrounds).

**Table 1:** Root nodule bacteria strains used in the Vavilov centers of diversity (Proposal ID: 231) and GEBA-RNB (Proposal ID: 882) genome sequencing projects

**Genus**	**Species**	**Strain**	**Country of isolation**	**Original host**
*Azorhizobium*	*Azorhizobium doebereinerae*	UFLA1-100^T^	Brazil	*Sesbania virgata*
*Bradyrhizobium*	*Bradyrhizobium elkanii*	USDA 76^T^	USA	*Glycine max*
*Bradyrhizobium*	*Bradyrhizobium elkanii*	USDA 94	USA	*Glycine max*
*Bradyrhizobium*	*Bradyrhizobium elkanii*	USDA 3259	USA	*Phaseolus lunatus*
*Bradyrhizobium*	*Bradyrhizobium elkanii*	USDA 3254	USA	*Phaseolus acutifolius*
*Bradyrhizobium*	*Bradyrhizobium sp.*	WSM1741	Australia	*Rhynchosia minima*
*Bradyrhizobium*	*Bradyrhizobium sp*	USDA 4	USA	*Glycine max*
*Bradyrhizobium*	*Bradyrhizobium japonicum*	*USDA 6^T^	Japan	*Glycine max*
*Bradyrhizobium*	*Bradyrhizobium japonicum*	USDA 38	Japan	*Glycine max*
*Bradyrhizobium*	*Bradyrhizobium sp.*	*USDA 122	USA	*Glycine max*
*Bradyrhizobium*	*Bradyrhizobium sp.*	USDA 123	USA	*Glycine max*
*Bradyrhizobium*	*Bradyrhizobium sp.*	USDA 124	USA	*Glycine max*
*Bradyrhizobium*	*Bradyrhizobium sp.*	USDA 135	USA	*Glycine max*
*Bradyrhizobium*	*Bradyrhizobium sp.*	WSM1743	Australia	*Indigofera sp.*
*Bradyrhizobium*	*Bradyrhizobium sp.*	*WSM2254	Australia	*Acacia dealbata*
*Bradyrhizobium*	*Bradyrhizobium sp.*	WSM2793	South Africa	*Rhynchosia totta.*
*Bradyrhizobium*	*Bradyrhizobium sp*.	Ai1a-2	Costa Rica	*Andira inermis*
*Bradyrhizobium*	*Bradyrhizobium sp*.	ARR65	Australia	*Stylosanthes viscosa*
*Bradyrhizobium*	*Bradyrhizobium sp*.	*CB756	Zimbabwe	*Macrotyloma africanum*
*Bradyrhizobium*	*Bradyrhizobium sp*.	Cp5.3	Panama	*Centrosema pubescens*
*Bradyrhizobium*	*Bradyrhizobium sp*.	EC3.3	Panama	*Erythrina costaricensis*
*Bradyrhizobium*	*Bradyrhizobium sp*.	Th.b2	USA	*Amphicarpaea bracteata*
*Bradyrhizobium*	*Bradyrhizobium sp*.	TV2a.2	Panama	*Tachigali versicolor*
*Bradyrhizobium*	*Bradyrhizobium sp*.	*USDA 3384	Brazil	*Crotalaria paulina*
*Bradyrhizobium*	*Bradyrhizobium sp*.	*WSM471	Australia	*Ornithopus pinnatus*
*Bradyrhizobium*	*Bradyrhizobium sp*.	WSM1253	Greece	*Ornithopus compressus*
*Bradyrhizobium*	*Bradyrhizobium sp*.	WSM1417	Chile	*Lupinus sp.*
*Bradyrhizobium*	*Bradyrhizobium sp*.	WSM2783	South Africa	*Leobordea carinata*
*Bradyrhizobium*	*Bradyrhizobium sp*.	WSM3983	Australia	*Kennedia coccinea*
*Bradyrhizobium*	*Bradyrhizobium sp*.	WSM4349	USA	*Syrmatium glabrum*
*Burkholderia*	*Burkholderia dilworthii*	WSM3556^T^	South Africa	*Lebeckia ambigua*
*Burkholderia*	*Burkholderia mimosarum*	STM3621	French Guiana	*Mimosa pudica*
*Burkholderia*	*Burkholderia mimosarum*	LMG 23256^T^	Taiwan	*Mimosa pigra*
*Burkholderia*	*Burkholderia sprentiae*	WSM5005^T^	South Africa	*Lebeckia ambigua*
*Burkholderia*	*Burkholderia symbiotica*	JPY347	Brazil	*Mimosa cordistipula*
*Burkholderia*	*Burkholderia tuberum*	WSM4176	South Africa	*Lebeckia ambigua*
*Burkholderia*	*Burkholderia* sp.	JPY251	Brazil	*Mimosa velloziana*
*Burkholderia*	*Burkholderia* sp.	JPY366	Brazil	*Mimosa misera*
*Burkholderia*	*Burkholderia* sp.	UYPR1.413	Uruguay	*Parapiptadenia rigida*
*Burkholderia*	*Burkholderia* sp.	WSM2230	Australia	*Kennedia coccinea*
*Burkholderia*	*Burkholderia* sp..	WSM2232	Australia	*Gastrolobium capitatum*
*Cupriavidus*	*Cupriavidus taiwanensis*	STM6018	French Guiana	*Mimosa pudica*
*Cupriavidus*	*Cupriavidus taiwanensis*	STM6070	New Caledonia	*Mimosa pudica*
*Cupriavidus*	*Cupriavidus* sp.	AMP6	USA	*Mimosa asperata*
*Cupriavidus*	*Cupriavidus* sp.	UYPR2.512	Uruguay	*Parapiptadenia rigida*
*Ensifer*	*Ensiferar boris*	LMG 14919^T^	Sudan	*Prosopis chilensis*
*Ensifer*	*Ensifer medicae*	Di28	Sardinia	*Medicago arabica*
*Ensifer*	*Ensifer medicae*	WSM244	Iraq	*Medicago polymorpha*
*Ensifer*	*Ensifer medicae*	*WSM1115	Greece	*Medicago polymorpha*
*Ensifer*	*Ensifer medicae*	WSM1369	Sardinia	*Medicago sphaerocarpos*
*Ensifer*	*Ensifer medicae*	WSM4191	Australia	*Melilotus siculus*
*Ensifer*	*Ensifer meliloti*	*4H41	Tunisia	*Phaseolus vulgaris*
*Ensifer*	*Ensifer meliloti*	CIAM1775	Kazakhstan	*Medicago lupulina*
*Ensifer*	*Ensifer meliloti*	GVPV12	Canary Islands (Spain)	*Phaseolus vulgaris*
*Ensifer*	*Ensifer meliloti*	Mlalz-1	Canary Islands (Spain)	*Medicago laciniata*
*Ensifer*	*Ensifer meliloti*	MVII-I	Germany	*Medicago sativa*
*Ensifer*	*Ensifer meliloti*	*RRI128	Australia	*Medicago truncatula*
*Ensifer*	*Ensifer meliloti*	WSM1022	Greece	*Medicago orbicularis*
*Ensifer*	*Ensifer* sp.	BR816	Brazil	*Leucaena leucocephala*
*Ensifer*	*Ensifer* sp.	PC2	India	*Prosopis cineraria*
*Ensifer*	*Ensifer* sp.	TW10	India	*Tephrosia wallichii*
*Ensifer*	*Ensifer* sp.	USDA 6670 (CC 2017)	Australia	*Medicago sativa*
*Ensifer*	*Ensifer* sp.	WSM1721	Australia	*Indigofera sp.*
*Mesorhizobium*	*Mesorhizobium ciceri*	CMG6	Tunisia	*Cicer arietinum*
*Mesorhizobium*	*Mesorhizobium ciceri*	WSM4083	Canary Islands (Spain)	*Bituminaria bituminosa*
*Mesorhizobium*	*Mesorhizobium loti*	CJ3sym	New Zealand	*Lotus corniculatus*
*Mesorhizobium*	*Mesorhizobium loti*	NZP2037	New Zealand	*Lotus divaricatus*
*Mesorhizobium*	*Mesorhizobium loti*	R7A	New Zealand	*Lotus corniculatus*
*Mesorhizobium*	*Mesorhizobium loti*	R88b	New Zealand	*Lotus corniculatus*
*Mesorhizobium*	*Mesorhizobium loti*	USDA 3471^T^	New Zealand	*Lotus corniculatus*
*Mesorhizobium*	*Mesorhizobium* sp.	WSM1293	Greece	*Lotus sp.*
*Mesorhizobium*	*Mesorhizobium* sp.	WSM2561	South Africa	*Lessertia diffusa*
*Mesorhizobium*	*Mesorhizobium* sp.	WSM3224	South Africa	*Otholobium candicans*
*Mesorhizobium*	*Mesorhizobium* sp.	WSM3626	South Africa	*Lessertia diffusa*
*Methylobacterium*	*Mesorhizobium* sp.	WSM2598	South Africa	*Listia bainesii*
*Microvirga*	*Microvirga lotononidis*	WSM3557^T^	Zambia	*Listia angolensis*
*Microvirga*	*Microvirga lupini*	Lut6^T^	USA	*Lupinus texensis*
*Rhizobium*	*Rhizobium giardinii*	H152^T^	France	*Phaseolus vulgaris*
*Rhizobium*	*Rhizobium leguminosarum bv. Phaseoli*	4292	UK	*Phaseolus vulgaris*
*Rhizobium*	*Rhizobium leguminosarumbv. Phaseoli*	FA23	Poland	*Phaseolus vulgaris*
*Rhizobium*	*Rhizobium leguminosarum bv. Trifolii*	*CB782	Kenya	*Trifolium semipilosum*
*Rhizobium*	*Rhizobium leguminosarum bv. Trifolii*	CC278f	USA	*Trifolium nanum*
*Rhizobium*	*Rhizobium leguminosarum bv. Trifolii*	*CC283b	Russia	*Trifolium ambiguum*
*Rhizobium*	*Rhizobium leguminosarum bv. Trifolii*	SRDI565	Australia	*Trifolium subterraneum*
*Rhizobium*	*Rhizobium leguminosarum bv. Trifolii*	SRDI943	Australia	*Trifolium subterraneum*
*Rhizobium*	*Rhizobium leguminosarum bv. Trifolii*	*TA1	Australia	*Trifolium subterraneum*
*Rhizobium*	*Rhizobium leguminosarum bv. Trifolii*	WSM597	Uruguay	*Trifolium pallidum*
*Rhizobium*	*Rhizobium leguminosarum bv. Trifolii*	WSM1689	Greece	*Trifolium uniflorum*
*Rhizobium*	*Rhizobium leguminosarum bv. Trifolii*	WSM2012	Ethiopia	*Trifolium ruepellianum*
*Rhizobium*	*Rhizobium leguminosarum bv. Trifolii*	WSM2297	South Africa	*Trifolium africanum*
*Rhizobium*	*Rhizobium leguminosarum bv. Viciae*	248	UK	*Vicia faba*
*Rhizobium*	*Rhizobium leguminosarum bv. Viciae*	128C53	UK	*Pisum sativum*
*Rhizobium*	*Rhizobium leguminosarum bv. Viciae*	GB30	Poland	*Pisum sativum*
*Rhizobium*	*Rhizobium leguminosarum bv. Viciae*	Ps8	UK	*Pisum sativum*
*Rhizobium*	*Rhizobium leguminosarum bv. Viciae*	TOM	Turkey	*Pisum sativum*
*Rhizobium*	*Rhizobium leguminosarum bv. Viciae*	UPM1131	Italy	*Pisum sativum*
*Rhizobium*	*Rhizobium leguminosarum bv. Viciae*	UPM1137	Italy	*Pisum sativum*
*Rhizobium*	*Rhizobium leguminosarum bv. Viciae*	Vc2	UK	*Vicia cracca*
*Rhizobium*	*Rhizobium leguminosarum bv. Viciae*	VF39	Germany	*Vicia faba*
*Rhizobium*	*Rhizobium leguminosarum bv. Viciae*	Vh3	UK	*Vicia hirsuta*
*Rhizobium*	*Rhizobium leguminosarum bv. Viciae*	WSM1455	Greece	*Vicia faba*
*Rhizobium*	*Rhizobium leguminosarum bv. Viciae*	WSM1481	Greece	*Vicia faba*
*Rhizobium*	*Rhizobium leucaenae*	*USDA 9039^T^	Brazil	*Phaseolus vulgaris*
*Rhizobium*	*Rhizobium mesoamericanum*	STM6155	New Caledonia	*Mimosa pudica*
*Rhizobium*	*Rhizobium mongolense*	USDA 1844^T^	China	*Medicago ruthenica*
*Rhizobium*	*Rhizobium sullae*	*WSM1592	Italy	*Hedysarum coronarium*
*Rhizobium*	*Rhizobium tibeticum*	OR 191	USA	*Medicago sativa*

^T^type strain; *commercial inoculant.

### Organism growth and nucleic acid isolation

The international consortium, which consists of more than 34 experts in the field from 15 different countries, together with Culture Collections Centers in Australia and Belgium will be growing the 107 different RNB. Quality Control will be performed for all samples before shipping the DNA to the JGI. All samples from members of the consortium that are based in the US, will be sent to Dr Peter van Berkum in Washington DC, and all other samples will be quality controlled at the Centre for *Rhizobium* Studies, Murdoch University in Australia before shipping to the JGI. Scientists at the Centre for *Rhizobium* Studies have extensive experience in producing high quality DNA, a skill acquired as a result of a long collaboration with the JGI as is evidenced by collaborative publication [17-22].

### Sequencing approach

Most RNB strains are characterized by multipartite genomes, the size of which varies between 5-10 Mb, with an average G + C%age of 60-65%. We propose drafting of the 107 RNB genomes using Illumina, PacBio or Roche sequencing platforms. All genomes will be completed to at least the stage of high quality draft. As most RNB strains carry their symbiotic genes on plasmids or within mobile islands that can be integrated in different sites on the chromosome, accurate scaffolding information is important for separation of chromosomal and plasmid-borne genes of interest.

### Annotation and comparative analysis

The microbial genome annotation pipeline at the JGI has been scaled to handle hundreds of microbial genomes per month [23-25].

### Publication of analyzed genomes

As many genomes as possible that are of publication quality will be published in *Standards in Genomic Sciences *[26,27].

## The scientific questions we expect to answer

The genome sequences of RNB generated in this project will be used to identify the core genomes of different RNB species, as well as dispensable parts of species pangenomes and their distribution between strains from different locales and/or plant hosts. Symbiotically relevant sets of genes such as those participating in adhesion, biosynthesis of nodulation factors, SNF, energy metabolism and exopolysaccharide biosynthesis will be characterized in detail. This will include the genes’ evolutionary histories and genome dynamics, such as localization on plasmids or within genomic islands and relation to mobile genetic elements. Statistical analyses will be performed in order to identify genes and gene sets that correlate with host specificity, nodulation and SNF efficiency and with various environmental metadata such as edaphic and climatic constraints. Within RNB strains of the same species, but from different environmental sites and/or legume hosts, genes that are under selective pressure will be identified and characterized by analysis of synonymous and non-synonymous substitution rates.

These analyses will be informed by the comprehensive metadata that are available for each strain, including data on the strains’ collection site, host specificity, nodulation and SNF efficiency. Considerable efforts have been devoted to sourcing strains from different geographical locations in order to improve legume productivity across a range of environments, and the project takes advantage of the particularly well characterized RNB that have been sourced from several culture collections around the globe. Biogeographic considerations are particularly relevant to the RNB as their survival and persistence as soil saprophytes is dictated by environmental and edaphic constraints such as temperature, salinity, pH, and soil moisture and clay content [28].

This project will support systematic sequence-based studies of the RNB and contribute to our understanding of the biogeographic effects on the evolution of different rhizobial species, as well as the mechanisms determining the specificity and efficiency of nodulation and N_2_-fixation by RNB.

## The relevance of the project to problems of societal importance

The symbiotic nitrogen fixation by RNB is a significant asset for world agricultural productivity, farming economy and environmental sustainability. Large-scale agricultural use of highly effective N_2_-fixing legumes will be critical for sustainable food production for livestock and humans. Increased incorporation of SNF into agricultural systems reduces the requirement for inputs of economically and environmentally costly nitrogenous fertilizer. Currently, ~1–2% of the world's annual energy supply is used in the Haber-Bosch process to manufacture chemical N, at a cost of $US 6.8 billion pa. In addition, SNF significantly reduces greenhouse gas emissions compared to intensive agriculture practice, which requires large inputs of chemical N. SNF also benefits the environment by helping to reduce dry-land salinity, increase soil fertility, promote carbon sequestration and prevent eutrophication of waterways. Recent publications have also emphasized the importance of providing renewable sources of biofuels [29,30], and a detailed understanding of endosymbionts and SNF will aid this quest. *Pongamia pinnata*, for example, is a leguminous tree that is important for the biofuel industry and is nodulated by a *Bradyrhizobium* strain [31] that has been included for sequencing in this proposal.

Apart from their economic importance, RNB also represent a uniquely tractable biological system that can offer insights into the shared genetic mechanisms between fungal and bacterial root endosymbioses [32] and between intracellular pathogens and endocytosed RNB microsymbionts. The latter have been shown to share similar host-adapted strategies in their infection processes and adaptation to growth within the cytoplasm of a eukaryotic host [33,34]. An understanding of these mechanisms will facilitate the quest to extend N_2_-fixation to cereals, a goal which is being vigorously pursued and which has been described as essential for future sustainable food production [35].

## Conclusion

The legume-RNB symbiosis is one of the best-studied associations between microbes and eukaryotes, due to the economic and ecological importance of symbiotic nitrogen fixation. Targeting RNB for sequencing on the basis of firstly, phylogenetic diversity and secondly, isolation from taxonomically distinct host legumes growing in diverse biomes offers significant benefits. Previous RNB sequencing projects have tended to focus on a narrow range of model organisms. By setting a goal of maximizing the phylogenetic diversity of sequenced RNB strains, these projects, in keeping with the other members of the GEBA family of projects, aid the development of a phylogenetically balanced genomic representation of the microbial tree of life and allow for the large-scale discovery of novel rhizobial genes and functions. The chosen RNB strains are available to the global research community and are stored in culture collections that are dedicated to long-term storage and distribution. A wealth of experimental data and metadata is available for each strain, which will inform analyses to identify genes and gene sets that correlate with rhizobial adaptation to diverse biomes, to the nodule environments found in taxonomically distinct legume hosts and to the effectiveness of nitrogen fixation within these nodules. Moreover, the legume-RNB symbiosis is an excellent model system to study plant-bacterial associations, including symbiotic signaling, cell differentiation and the mechanisms of endocytosis. The sequenced RNB genomes will not only provide a greater understanding of legume-RNB associations, but can be used to gain insights into the evolution of N2-fixing symbioses and microbe-eukaryote interactions.

## Abbreviations

RNB: Root nodule bacteria; N_2_: Dinitrogen; N: nitrogen; SNF: Symbiotic nitrogen fixation; N_2_O: Nitrous oxide; GEBA: Genomic Encyclopedia for Bacteria and Archaea.

## Competing interests

The authors declare that they have no competing interests.

## Authors’ contributions

WR and JA supplied background information for this project, TR supplied DNA to the JGI, WR and JA drafted the paper, JWY and PN supplied figures and all other authors were involved in sequencing the genomes and/or editing the final paper. All authors read and approved the final manuscript.
